# A molecular cytogenetic perspective on chromosome biology and crop improvement

**DOI:** 10.1002/tpg2.70126

**Published:** 2025-09-27

**Authors:** Bikram S. Gill

**Affiliations:** ^1^ Wheat Genetics Resource Center, Department of Plant Pathology Kansas State University Manhattan Kansas USA

## Abstract

The age of molecular cytogenetic analysis of crop plants dawned in the late 1960s and early 1970s with new advances in the identification of somatic chromosomes by C‐banding and fluorescence in situ hybridization concurrent with advances in DNA cloning, sequencing, and mapping. In this perspective article dedicated to Ronald Phillips, I review the contributions of molecular cytogenetic research to chromosome biology and crop improvement. I argue that molecular cytogenetics and wide hybridization (intergeneric and interspecific hybridization followed by introgressive breeding) will continue to play a key role in developing climate‐resilient crop germplasm. However, this will happen only if the lack of investment and retrenchment of faculty engaged in molecular cytogenetics is reversed across US land‐grant universities.

## Core Ideas

- Molecular cytogenetic has provided new insights into chromosome biology.
- Centromeres are mutable, movable, and amenable to de novo synthesis.
- McClintock proposed the theory of dynamic genome.
- Molecular cytogenetics is used to monitor genetic transfers in wide hybridization, and crop improvement.

## Plain Language Summary

Chromosomes are the physical basis of Mendelian heredity and plant breeding. New advances in chromosome manipulation of wide hybrids hold great promise for crop improvement. More investment in chromosome research is needed for realizing the potential of wide hybridization in crop improvement.

**Abbreviations**
BAC: bacterial artificial chromosome
bfb: breakage‐fusion‐bridge
eccDNA: extrachromosomal circular DNA
FISH: fluorescence in situ hybridization
WSMV: wheat streak mosaic virus

## 1 INTRODUCTION

**FIGURE 1:**
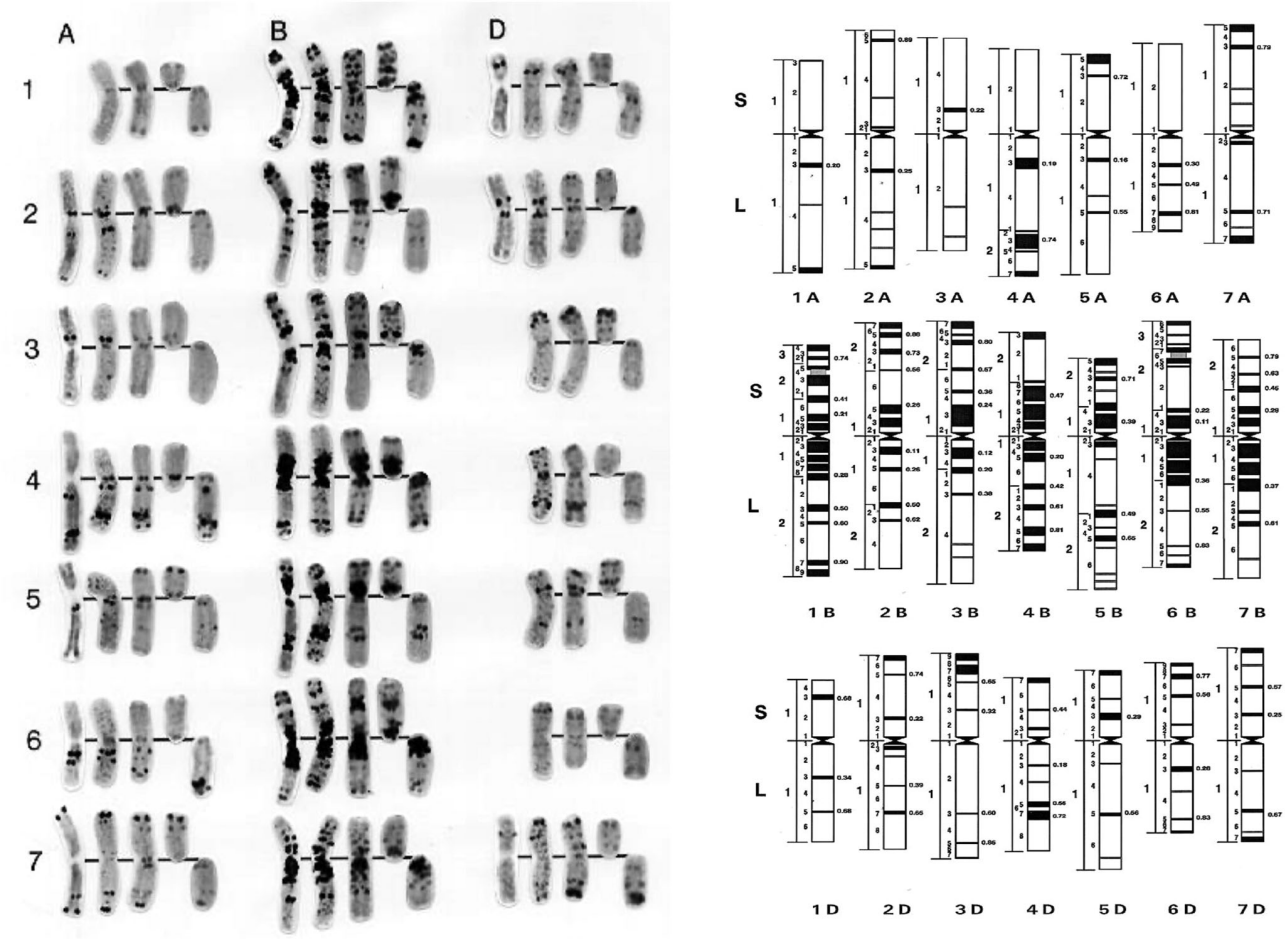
Standard C‐banded karyotype of common or bread wheat (*Triticum aestivum* L.; 2*n* = 6*x* = 42, genomes A, B, and D). Left panel. The visualization of heterochromatic (dark bands) and euchromatic (light staining) regions in somatic chromosomes and telosomes of wheat permits individual identification of the 21 chromosomes. Right panel: Diagrammatic representation, nomenclature system, and cytological maps of the 21 chromosomes of wheat for fine mapping of traits, DNA sequences, and genes. Reprinted with permission from B. S. Gill et al. (1991).

**FIGURE 2:**
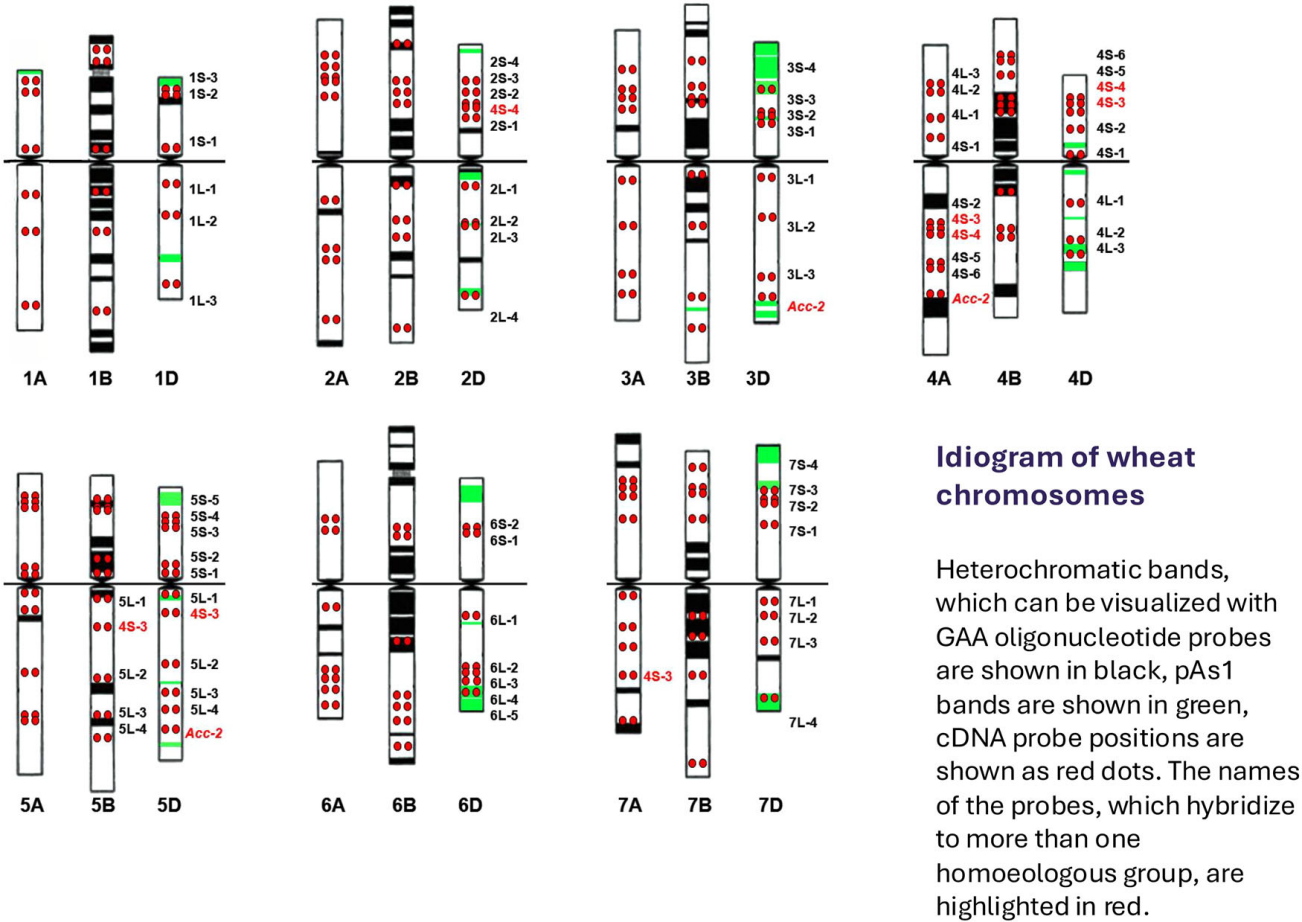
Standard fluorescence in situ hybridization (FISH) cytogenetic karyotype of common wheat. Molecular cytogenetic maps of the 21 chromosomes of wheat are diagnostic for individual chromosome identification and constructed based on FISH signals using repetitive DNA (GAA oligo and pAs1 for visualization of heterochromatin) and single copy DNA probes for rapid synteny and collinearity analysis of homoeologous chromosomes of wheat and wheat relatives. Reprinted with permission from Danilova et al. (2014).

**FIGURE 3:**
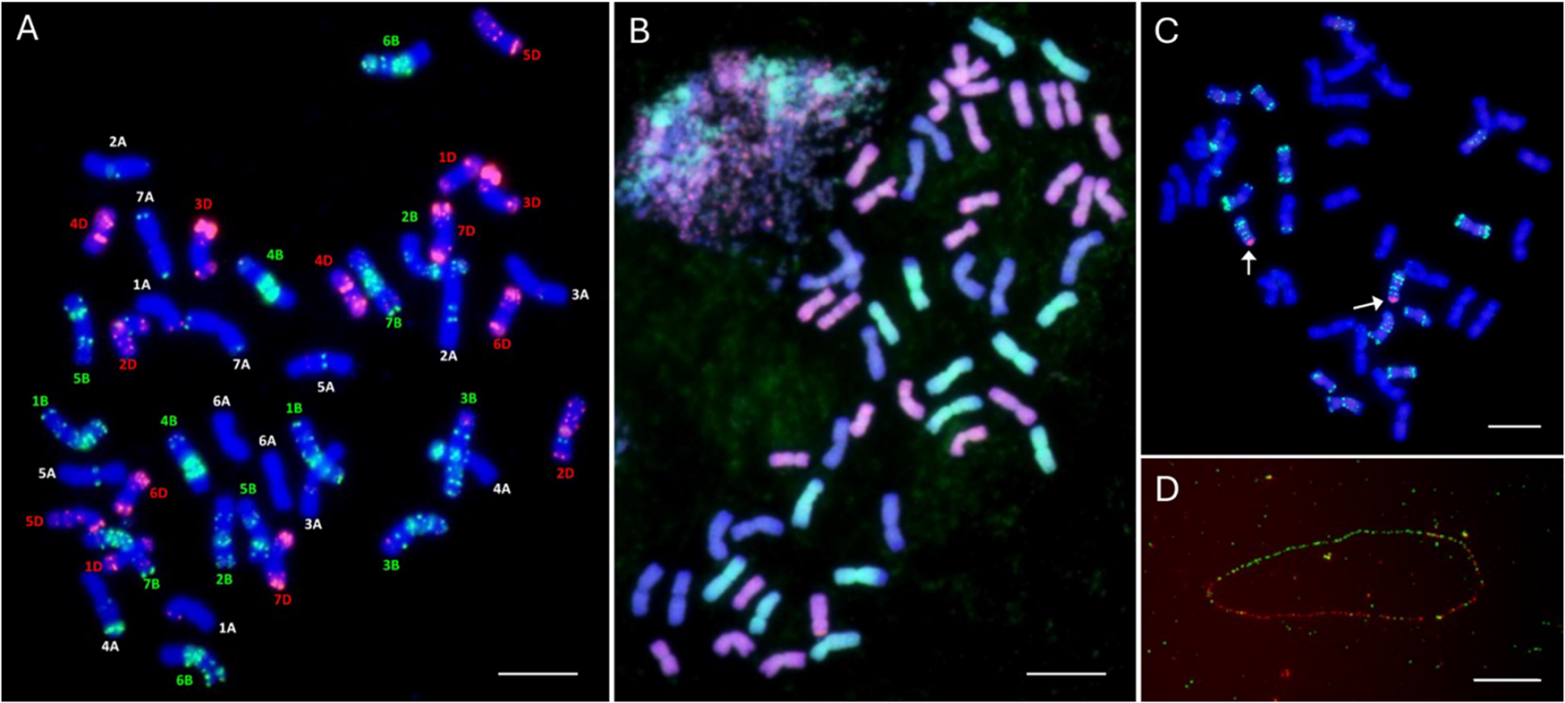
Fluorescence in situ hybridization (FISH) applications in chromosome and genome analysis. (A) Identification of individual wheat chromosomes by FISH using GAA and pAs1 oligonucleotide probes. Oligonucleotide probes GAA (24 nt) and pAs1 (26 nt) were synthesized by Integrated DNA Technologies, with a fluorochrome attached to the 5′ end; FITC (green) for oligo GAA and Cy3 (red) for pAs1 (Danilova et al., 2012). (B) Discrimination of wheat sub‐genomes A (green), B (blue), and D (red) by chromosome painting using total genomic DNA as probes in an octoploid wheat (AABBDDD^t^D^t^) derived from chromosome doubling of wheat (ABD) × *Aegilops tauschii* L. (D^t^) F1 hybrid (ABDD^t^). Total genomic DNA of diploid wheat, *Triticum monococcum* L. (A^m^A^m^) and *Ae. tauschii* was labeled with biotin‐16‐dUTP and digoxigenin‐11‐dUTP, respectively. Biotin‐ and digoxigenin‐labeled DNAs were detected with FITC (green) and Cy3 (red), respectively; chromosomes were counterstained with 4′,6‐diamidino‐2‐phenylindole (DAPI) (blue) (Gill & Koo, unpublished data). (C) Detection of a wheatgrass (*Thinopyrum intermedium* (Host) Barkworth & D.R. Dewey) chromosome segment (solid red) by chromosome painting carrying *Wsm1* (wheat streak mosaic) resistance gene (indicated by arrows) transferred to wheat chromosome 4DS, identified using pAs1 probe (green) (Friebe et al., 2009). (D) Fiber‐FISH of extrachromosomal circular DNA (eccDNA; 399,435 bp) containing the EPSPS gene conferring glyphosate resistance in *Amaranthus palmeri* S. Watson. Six bacterial artificial chromosome (BAC) clones specific to eccDNA were used: BAC 8H14, BAC 1G15, and BAC 13C09 were labeled in green, and BAC 22F22, BAC 23A10, and BAC 3A06 were labeled in red (Koo et al., 2023). Bars = 10 µm.

Ron Phillips was, first and foremost, a cytogeneticist. Soon after joining my first faculty position with the University of Florida in 1978, I travelled to Ron Phillips's laboratory at the University of Minnesota to learn in situ hybridization, a technique that essentially allows mapping of DNA sequences, repetitive or genic, directly onto the chromosomes on a glass slide. Ron and his wife, Judy, came to receive me at the airport, but somehow, we missed each other. I called when Ron and Judy had just arrived back home, and they promptly came back to pick me up! I never forgot their wonderful hospitality. Ron became my mentor and friend throughout my academic career. During my brief stay in St. Paul, I also spent time with Charley Burnham (one of the fabulous five corn geneticists, including Barbara McClintock, George Beadle, Marcus Rhoades, and Lewis Stadler, who trained with Rollins Emerson at Cornell University in the late 1920s and early 1930s) working on a manuscript describing a tester set of translocations in tomato (*Lycopersicon esculentum* L.) (B. S. Gill et al. 1980), a collaboration I began when I was a graduate student with Charley Rick at the University of California in Davis. In those days, corn (*Zea mays* L.) and tomato were great cytogenetic models because individual chromosomes could be identified at the pachytene stage of meiosis, first demonstrated by McClintock in corn (McClintock, 1929). In most plants, including our major crop plants, individual chromosome identification was impossible. It would soon change in the early 1970s, when individual somatic chromosomes from readily available root meristems were cytogenetically identified by C‐banding in rye (*Secale cereale* L. (B. S. Gill & Kimber, 1974a), and wheat (B. S. Gill & Kimber, 1974b; B. S. Gill et al., 1991; Figure 1) and other plants with large chromosomes. I moved to Kansas State University in 1979, and new advances in fluorescence in situ hybridization (FISH) (Jiang, 2019; Jiang & Gill, 1994; Jiang & Gill, 2006; Rayburn & Gill, 1985) launched the field of molecular cytogenetics defined as integrated molecular and cytogenetic analysis of chromosome structure, function, behavior, evolution, engineering, and diagnostics, applicable to all crop plants and organisms, irrespective of chromosome size or number (B. S. Gill, 1993; Figures 2 and 3). This perspective article in molecular cytogenetics is dedicated to the memory and academic contributions of Ron Phillips. I also had the pleasure of working with many of Ron's students, including Roberto Tuberosa (Tuberosa et al., 2002), who is editing this dedication issue of *Plant Genome*. I should state at the outset that this is not a compressive review of molecular cytogenetics but rather highlights research from our own laboratory to illuminate the power of the molecular cytogenetic approach in unraveling certain aspects of chromosome biology and crop improvement.

## 2 MOLECULAR CYTOGENETIC EXPLORATIONS INTO CHROMOSOME BIOLOGY

Classically, a linear chromosome consists of a centromere (that organizes a kinetochore for capturing microtubules for chromosome movement during cell division) that divides it into short and long arms differentiated into dark‐staining heterochromatic and light‐staining euchromatic regions capped by telomeres at each end. In different organisms, chromosomes can be individually identified based on total length, arm ratio, and the pattern of distribution of heterochromatic knobs or bands. Molecular cytogenetic research has provided unique insights in the structure and function of these classical cytological landmarks.

**Core Ideas**

- Molecular cytogenetic has provided new insights into chromosome biology.
- Centromeres are mutable, movable, and amenable to de novo synthesis.
- McClintock proposed the theory of dynamic genome.
- Molecular cytogenetics is used to monitor genetic transfers in wide hybridization, and crop improvement.

### 2.1 Centromeres are mutable, movable, and amenable to de novo synthesis

While working on the tomato tester set of translocations, I determined the translocation breakpoints by analyzing quadrivalents at pachytene and classified the breakpoints to heterochromatic or euchromatic regions or centromeres. Burnham promptly pointed out that we do not know if centromeres are divisible and, instead, we changed the wording to “centromeric regions” throughout the manuscript. A serendipitous discovery on a collaboration project with Rod Wing's lab on checking the quality of the first plant bacterial artificial chromosome (BAC) library in *Sorghum bicolor* (L.) Moench (Woo et al., 1994), my student Jiming Jiang discovered that certain BACs were exclusively localized to the centromeres. Some of the subcloned sequences from one sorghum BAC were conserved in the centromeres of all grasses, indicating a probable functional centromeric DNA element (Jiang et al., 1996). We documented hybrid centromeres in wheat–rye (*Secale cereale* L.) translocation chromosomes using centromere‐specific probes, indicating that centromere do, in fact, break (P. Zhang et al., 2001). We found that telosomic chromosomes in wheat had breaks in the centromeres but were stabilized by de novo addition of telomeric sequences (Werner, Kota, et al., 1992). Usually dicentric chromosomes are unstable, but molecular cytogenetic analysis of a stable dicentric chromosome in wheat provided support for the mounting evidence that centromere function is under epigenetic control and specified by the recruitment of a variant histone CENH3 that is specific to the centromeres (W. Zhang et al., 2010). Basic research is often impactful in practical applications and defective CENH3 histones are unable to load onto centromeres, and this has been exploited in haploid induction in many crop plants (Ravi & Chan, 2010). Artificial centromeres have been synthesized using the LexA‐CENH3 fusion protein to recruit native Centromeric Histone H3 (CENH3) to long arrays of LexO repeats on a maize (*Zea mays* L.) chromosome arm that was transmitted to the progeny, opening the possibility of artificial chromosomes as potential payload vehicles for crop improvement (Dawe et al., 2023).

### 2.2 Heterochromatin consists of tandem repeats of DNA families and is prone to duplications and deletions facilitating rapid adaptive evolution

I visualized massive heterochromatic and highly polymorphic C‐bands in rye while working on the first rye cytogenetic karyotype (B. S. Gill & Kimber, 1974a) and cytogenetic identification of rye trisomics (Zeller et al., 1977). In the 1970s and early 1980s, plant and animal genomes were shown to contain a massive amount of tandemly repeated DNA and repeat families of many megabases, with repeat elements varying from a few base pairs to few kilobases in length. Most of these repeat elements were localized to heterochromatic regions, including our first report on a versatile DNA probe element pAs1 (Figure 2) and the technique we used to identify the D‐genome chromosomes of wheat (Rayburn & Gill, 1986; see Figure 2). In fact, the C‐banding technique is practically extinct, as cytogeneticists practicing C‐banding have retired or are no longer with us and, instead, a cocktail of repeat DNA probes and single‐copy probes is used for cytogenetic identification of individual chromosomes (Danilova et al., 2014; Figures 2 and 3). I do not want to delve into the massive literature on heterochromatin structure, function, behavior, and the notion that it is a junkyard component of eukaryotic genomes. However, we have documented several examples of the adaptive value of heterochromatic size polymorphism due to frequent duplication deletions presumably due to ectopic recombination among repeat families. We know that heterochromatin is loaded with repetitive DNA, but it does contain genes, although at a much lower frequency than euchromatin. We conducted molecular cytogenetic analysis to study nature of herbicide resistance in several crop weeds. Using in situ hybridization, we localized an herbicide target gene to a small heterochromatic knob in susceptible plants and amplified gene copies to a large heterochromatic knob in the same chromosome location in herbicide‐resistant plants (Koo et al., 2025). In another crop weed, the common waterhemp (*Amaranthus tuberculatus* (Moq.) Saur), the target gene was localized to the pericenter heterochromatic region and escaped the chromosome as a ring chromosome that underwent frequent breakage‐fusion‐bridge (bfb) cycles, amplifying the target gene and leading to rapid evolution of glyphosate resistance (Koo, Jugulam, et al., 2018). Thus, apart from other biological functions, such as silencing of transposable elements, heterochromatin facilitates rapid adaptive response by gene copy number polymorphisms.

### 2.3 McClintock's healing telomeres and misbehaving telomeres

In the 1940s, McClintock demonstrated that broken chromosomes were unstable and special structures called telomeres must protect the chromosome ends. We now know that telomere structure is conserved among all eukaryotic organisms and consists of a simple polymer of (TTTAGGG)n bases with slight alterations. However, as it often happens in biology, there are always exceptions. *Allium* species were found to lack the canonical telomeric sequences (Fuchs et al., 1995), and instead, it has been shown recently that *Allium* genus telomeres are comprised of (CTCGGTTATGGG)n polymers, and in common with canonical telomeres, they are also synthesized by telomerase (Fajkus et al., 2016). We isolated hundreds of deletion stocks in wheat (Endo & Gill, 1996), and extensive cytogenetic mapping indicated that most of the deletions were simple deficiencies. Our molecular cytogenetic studies confirmed McClintock's conjecture, and the deletion chromosomes, including Sears's telosomic stocks, were healed by the de novo addition of telomeric sequences (Werner, Kota, et al., 1992). Chromosomes perform a ballet dance during cell division, driven by the telomeres, which are clustered together at the periphery of the nuclear envelop during prophase of meiosis and also mitosis, as was reported by Jiming Jiang's lab (Dong & Jiang, 1998). This clustering provides opportunities for ectopic recombination and gene conversion leading to copy number variation in the telomeric regions (Freitas‐Junior et al., 2000; our unpublished results). I suppose that any missteps during this ballet dance, for example, telomeres ending up in the centromeric territory (at the opposite end) and vice versa, may lead to telomere ends of one chromosome to undergo an ectopic recombination event with the centromere of another chromosome leading to dysploidy and reduction in chromosome number as reported in the Triticeae (Luo et al., 2009). Of course, the bouquet stage also provides opportunities for the telomeres of two different chromosomes to recombine or fuse leading to dicentric chromosomes launching McClintock's bfb cycle, genomic earthquake, and chromosome/genome reorganization. We documented a dicentric chromosome arising from the fusion of a wheat chromosome and an alien telocentric chromosome, which presumably underwent bfb cycle and recovered a compensating wheat–alien chromosome translocation in plant progenies specifying resistance to wheat streak mosaic virus (WSMV) (Liu, Seifers, et al., 2011). Finally, synapsis among homologous chromosomes is initiated at the chromosome ends, presumably facilitated by telomere movements during the bouquet stage of meiosis and may explain the high rates of recombination at chromosome ends with profound implications for breeding and biology (see Section 2.4).

### 2.4 Euchromatin: location matters

Compared to relatively inert heterochromatin, euchromatin is the active portion of the chromatin but its position along the telomere‐centromere axis profoundly affects the frequency as well as fate of the genes in the different euchromatic regions. Our first‐generation, deletion‐bin‐based, comparative cytogenetic maps showed a dramatic suppression of recombination in the proximal centromeric regions and very high recombination in the very distal telomeric chromosomal regions (Werner, Endo, et al., 1992), which was correlated with a high density of genes in the distal regions (Akhunov et al., 2003; K. S. Gill et al., 1996). My student Deven See used to say that Darwin's workshop was located at the chromosome ends as he found a high frequency of novel, unique‐copy DNA sequences at the chromosome ends (See et al., 2006). Most of the highly evolving resistance genes were localized at the chromosome ends (L. L. Qi et al., 2004). During the cloning of the leaf rust resistance gene *Lr21*, which is present at the very tip of the short arm of chromosome 1D, we documented a gene conversion event within the *Lr21* locus leading to susceptibility (Huang et al., 2003). We also recovered a resistance allele from an intragenic recombination event involving susceptible alleles at the *Lr21* locus (Huang et al., 2009). In the weed *Kochia scoparia* (L.) Schrad, a glyphosate target gene is in a high recombination region at the end of a chromosome, and this species evolved resistance to glyphosate by amplification of the target gene through multiple rounds of unequal recombination and tandem duplications as documented by fiber‐FISH (Jugulam et al., 2014). Does this mean that genes in recombination‐suppressed regions are in evolutionary stasis? Not really, as some of our recent molecular cytogenetic research brings out McClintock's fluid genome in full focus in the next section.

### 2.5 McClintock's fluid genome gets real

Mendelian genetics, modern breeding, and evolutionary theories are based on the notion that (a) genes are trapped in chromosomes in perpetuity, (b) the genome (chromosome complement) is faithfully reproduced by mitosis, (c) new alleles arise by random mutations, (d) recombination during meiosis produces new allelic combination (haplotypes), and (e) the collection of genotypes from Mendelian heredity is the subject of improved breeding following selection in agriculture and new biotypes and species in nature. McClintock documented transposable elements that can escape and reinsert in different chromosome locations, and dicentric and ring chromosomes that can reorganize genomes (reviewed in McClintock [1978]). McClintock painted a picture of dynamic genome and postulated innate genetic elements that are capable of response under stress (McClintock, 1984). We documented an innate extrachromosomal circular DNA (eccDNA) element (replicon) in the crop weed Palmer Amaranth (*Amaranthus palmeri*) that escaped the control of mitosis, providing a vehicle for target gene amplification in dividing soma cells and mounting an acquired resistance response to glyphosate (Koo, Molin, et al., 2018; Figure 3D). The replicon eccDNA element is ∼400 kb, has 79 genes co‐opted from eight different chromosomes, and is an organelle capable of replication and anchoring to chromosomes for genetic transmission (Koo, Molin, et al., 2018; Molin et al., 2020). The Palmer Amaranth genome fashioned this natural genetic engineering element to survive herbicide onslaught in a mere span of 8 years following the introduction of Round‐Up ready crops in 1998. In fact, we now know that nuclear genomes are teeming with transitory eccDNA elements, and any sequence or gene can escape a chromosome, for example, through ectopic recombination among shared repeats (Møller et al., 2015). Telomeric, centromeric, and ribosomal DNA consist of tandemly repeated families, which frequently escape as eccDNA and insert at nonnative chromosome locations. McClintock's dynamic genome is real, capable of mounting rapid response, and has profound implications in agriculture, medicine, and nature of evolution and creation of biodiversity.

### 2.6 Genome sequencing, oligo‐FISHing, chromosome painting, and new frontiers of chromosome research

Genome sequencing has revolutionized biological research, including molecular cytogenetic research. One of the cumbersome parts of FISH was handling and sharing of cloned DNA probes. Our first wheat D‐genome diagnostic probe pAs1 plasmid was maintained and purified from bacterial cultures, and plasmid DNA was shipped around the world for sharing, obviously a time‐consuming and costly exercise. But now we have synthesized oligo‐probes for pAs1 and other repeats (Figure 2). The oligo‐FISH probes can be designed from simple or complex repeats or single‐copy genes online from sequenced genomes, synthesized and labeled with florescence tags and used for FISH for chromosome barcoding, painting, and other applications in basic and applied research (for an authoritative review, see Jiang [2019]). Individual chromosome painting for small genomes using cocktails of BAC‐FISH elucidated chromosome changes in structure and number during speciation (Lysak et al., 2001, 2006). Now gene‐based oligos can be synthesized and used for chromosome painting (Jiang, 2019). Amazingly, gene‐based SNPs were used to design haplotype‐based oligo FISH assays to visualize meiotic crossovers in a maize hybrid (Martins et al., 2019). I have barely scratched the surface of new developments in cytogenetics, especially in animal cytogenetics, where techniques and assays are available for live imaging and analysis of chromosome structure and behavior in dividing cells (for review, see Shapiro [2021]). As I mentioned earlier, there is amazing diversity in chromosome structure and number in diverse organisms despite the constraints imposed by mitosis and meiosis (Lysak, 2022; Schubert & Lysak, 2011). From cancer and plant cytogenetics research, we are learning about chromoanagenesis (birthing of new chromosomes) by chromothripsis (massive shattering and rejoining of chromosomes in a single mitotic event), chromoplexy (weaving involving several chromosomes) and, of course, the classical chromosome bfb cycles that can drive rapid chromosome reorganization and macroevolution (Guo et al., 2023; Houben et al., 2025; Pellestor & Gatinos, 2020; Shapiro, 2021). Soon, these basic findings will find their application in crop improvement enterprise.

### 2.7 Molecular cytogenetics, wide hybridization (intergeneric and interspecific hybridization followed by introgressive breeding), and crop improvement

Molecular cytogenetics and wide hybridization had a huge impact in crop improvement as well (B. S. Gill, 2022; B. S. Gill et al., 2006; B. S. Gill et al., 2019; Jiang et al., 1994). Our laboratory collaborated with cytogeneticists in the Great Plains region, the United States, and the world in the molecular cytogenetic analysis of wheat germplasm with useful traits (reviewed in Friebe et al. [1996]; B. S. Gill et al. [2006]). In a pioneering paper, we analyzed several X‐ray irradiated lines from crosses of wheat with wheatgrass and *Aegilops speltoides* L. (produced by Daryl Wells at South Dakota State University for resistance to WSMV and greenbug) by C‐banding and in situ hybridization (Friebe et al., 1991). From this complex material, we identified an agronomically desirable compensating translocation carrying the WSMV‐resistance gene *Wsm1*. It took us another 16 years to streamline the chromosome engineering methodology to isolate *Wsm1* recombinants (Koo et al., 2020; L. Qi et al., 2007). In Oklahoma, Sebesta was using X‐rays to transfer greenbug and Hessian fly resistance from rye into wheat. We documented the famous T1AL·1RS translocation in his Amigo wheat. We also documented Sebesta's rare feat of X‐ray‐induced chromosome engineering in the transfer of a small intercalary rye chromatin segment carrying Hessian fly resistance gene *H25* integrated into a wheat chromosome, which was featured on the cover of the journal *Chromosoma* (Mukai et al., 1993). One last and most notable example was chance hitting on a treasure trove of useful cryptic alien variation (untargeted spontaneous transfers) in wheat × *Aegilops geniculata* hybrids (produced by Dhaliwal at PAU‐Ludhiana); all derived from chromosome 5M^g^ for leaf and stripe rust resistance (Kuraparthy et al., 2007), stem rust resistance (Liu, Rouse, et al., 2011), and homoeologous pairing promotor genes for efficient recombination (Koo et al., 2016; Koo et al., 2020). In summary, we analyzed several dozen germplasm lines from all over the world, including material from our own wide hybridization program and documented a large number of novel genes that are now making an impact in production agriculture (http://www.k‐state.edu/wgrc/resources/genetic_resources/; Friebe et al., 1996; B. S. Gill et al., 2006).

### 2.8 Ron Phillips’ holy grail and unrealized promise of wide hybridization

From a molecular cytogenetics perspective, I want to briefly discuss the classic research of Ron Phillips and his colleague Howard Rines using wide hybridization to isolate a complete set of maize chromosome additions in the oat genome (Kynast et al., 2001). These materials were the “holy grail” for potential transfer of a complex trait, such as C4 photosynthesis, from a C4 maize plant to a C3 oat plant, among many other such physiological traits (Warzecha et al., 2023). As reviewed by Phillips’ group (Kynast et al., 2001; see also Warzecha et al. [2023]), the relevant maize C4 photosynthetic genes were expressed in the oat–maize lines and localized to several different chromosomes. Following our success in transferring and characterizing small, alien introgressions of rye into wheat (Mukai et al., 1993), I was convinced that similar experiments will be successful in introgressing maize photosynthetic genes into oats. Upon Rines's and Phillips's retirement, they graciously shared this material and blessed these planned experiments. It was rather a big task, needing a major investment of time and resources. Meanwhile, we were overwhelmed by our involvement in the wheat‐genome sequencing project. To my regret, we never could work on this project and share results with Ron. However, seeing the recent paper by Warzecha et al. (2023), I am glad that the dream of this holy grail of wide hybridization research on transferring complex physiological traits is still alive. If not by wide hybridization, other modern and highly precise genetic engineering tools such as CRISPR‐Cas9 also hold promise (Tuncel et al., 2025).

## 3 FUTURE PROSPECTS

The 20th century belonged to Barbara McClintock, Ronald Phillips, and many other cytogeneticists who made pioneering contributions to chromosome and genome research and crop improvement. When I began my career, every US land‐grant university had one dedicated cytogeneticist as a part of the crop improvement team. This generation of cytogeneticists has retired, and those positions have been closed or dedicated to other areas of research. As I have demonstrated, cytogenetics provides a front‐row view of chromosome structure and behavior in hundreds of dividing cells in real time. This research has provided unique insights into genome structure, function, and evolution with far‐reaching implications for crop improvement enterprise. Wide hybridization is a critical area of crop improvement research, and only a highly trained cytogeneticist can handle such material and search for nuggets in a mountain of dirt. My molecular cytogenetic perspective will be successful if this trend can be reversed. With fully sequenced genomes, molecular cytogenetics offers unique prospects for analyzing chromosome structure and behavior in wide hybrids and its manipulation in crop improvement. Adaptive complexes for climate‐resilient traits exist in crop plant relatives but they can only be transferred through wide hybridization, chromosome engineering, and analysis in well‐equipped molecular cytogenetic laboratories manned by competent cytogeneticists.

## AUTHOR CONTRIBUTIONS

**Bikram S. Gill**: Conceptualization; funding acquisition; investigation; writing—original draft; writing—review and editing.

## CONFLICT OF INTEREST STATEMENT

The author declares no conflicts of interest.
